# Exploring the effects of COLOSTRONONI on the mammalian gut microbiota composition

**DOI:** 10.1371/journal.pone.0217609

**Published:** 2019-05-31

**Authors:** Sabrina Duranti, Leonardo Mancabelli, Walter Mancino, Rosaria Anzalone, Giulia Longhi, Rosario Statello, Luca Carnevali, Andrea Sgoifo, Sergio Bernasconi, Francesca Turroni, Marco Ventura

**Affiliations:** 1 Laboratory of Probiogenomics, Department of Chemistry, Life Sciences, and Environmental Sustainability, University of Parma, Parma, Italy; 2 GenProbio srl Parma, Parma, Italy; 3 Stress Physiology Laboratory, Department of Chemistry, Life Sciences and Environmental Sustainability, University of Parma, Parma, Italy; 4 Microbiome Research Hub, University of Parma, Parma, Italy; Wageningen University, NETHERLANDS

## Abstract

COLOSTRONONI is a dietary supplement consisting of bovine colostrum and *Morinda citrifolia* fruit (Noni). In this study, we tested the capability of COLOSTRONONI to influence gut microbiota composition using an *in vivo* evaluation in rats. Furthermore, we analyzed the effect of COLOSTRONONI on the systemic inflammatory responses as well as on the gut permeability of the animals. Altogether, our analyses supported the concept of COLOSTRONONI as a natural food supplement that doesn't affect (neither negatively nor positively) gut microbiota homeostasis in healthy conditions. Moreover, COLOSTRONONI highlighted a lower effect in the expression of genes coding for IL-10, Il-12 and TNF-α response allowing us to hypothesize an immunomodulatory activity of this dietary supplement.

## Introduction

COLOSTRONONI is a new dietary supplement consisting of bovine colostrum and *Morinda citrifolia* fruit (Noni) [1]. Several scientific evidences suggested that bovine colostrum and noni fruit exert regulatory effects which prevent intestinal inflammation conditions and, consequently, are able to reduce the develop of inflammatory-based chronic systemic diseases [1].

Colostrum is the first milk collected from a lactating mammal gland after delivery. It represents a unique aspect to nutrition of newborns, in fact it is able to promote the development of microbial composition of gastrointestinal tract as well as the immune system through the acquisition of maternal immunoglobulins [2]. Bovine colostrum is rich in inflammatory cytokines including interleukins (IL-2, IL-1β, IL-6, IL-17) and immunoglobulins, such as IgA, IgM and IgG, tumor necrosis factor-α (TNF-α), interferon-γ, and other not antimicrobial compounds contributing to protect the organism against pathogenic microorganism infections [3, 4]. In addition, the bovine colostrum encompasses components involved in the innate immune system like antimicrobial peptides as well as lactoferrin and lactoperoxidases, displaying additive antibacterial effects [4, 5].

The *Morinda citrifolia* L., also known as Noni, is a plant producing more than 160 identified phytochemicals, which is native to the Indian Ocean and Polynesia and it is widely distributed around the world [6]. Biological compounds such as glycosides, polysaccharides, trisaccharide fatty acid esters, vitamins, and minerals have been isolated from noni fruits, roots, and leaves [7]. Recent studies revealed that juice made extract from the *M*. *citrifolia* fruit (Noni) possesses a broad range of immunomodulatory effects, including antibacterial, anti-inflammatory, anti-tumorigenesis, and antioxidant activities [7–11].

The intestinal barrier is able to regulate the absorption of nutrients and the exchange of molecules between the host and environmental [12]. Specifically, the inter-endothelial tight junctions (TJs) play a critical role in the permeability of the endothelial barrier [13]. In particular, TJs connect neighboring cells with each other to create a barrier that prevents pathogen infections as well as regulates paracellular diffusion of ions and solutes [14].

The aim of this study was the evaluation of the influence of COLOSTRONONI on the overall microbial biodiversity in the gut of rats, in order to identify a putative effect of this dietary supplement on specific bacterial groups. Furthermore, in this study we assessed the effect of COLOSTRONONI on the systemic inflammatory responses as well as on the gut permeability of rats.

## Material and methods

### Ethical statement

All experimental procedures and protocols involving animals were approved by the Veterinarian Animal Care and Use Committee of Parma University (number 370/2018) and conducted in accordance with the European Community Council Directives dated 22 September 2010 (2010/63/UE).

### Animal housing

Experiments involved three-month-old male wild-type Groningen rats (*Rattus norvegicus*), originally obtained from the University of Groningen (The Netherlands), and bred in animal facilities under standard conditions. After weaning, rats were housed in same sex sibling groups in rooms under humidity-(50 ± 10%) and temperature (22 ± 2°C) controlled conditions, a 12-h light–dark cycle (lights on at 7 a.m.), and with food and water available *ad libitum*.

### COLOSTRONONI description

COLOSTRONONI is a dietary supplement consisting of 30.56% of bovine colostrum and 11,11% of *Morinda citrifolia* fruit juice (Noni) (S1 Table). The manufacturing process flow sheet (Colostrononi 2205C02) is displayed in S1 Fig. The list of all ingredients is described in S1 Table. Moreover, the quality control is performed by Verdellino Laboratory, Italy and the number of analytic certificate was 201727122. The details concerning the quality check are described in S2 Table. The stability experiments revealed that the shelf life of COLOSTRONONI is 36 months (S3 Table). Moreover, the product was storaged in cool and dry place, protected from direct sunlight.

Finally, the chemical characterizations of Bovine Colostrum and *Morinda citrifolia* fruit juice are provided in S4 and S5 Tables.

### Experimental design of the *in vivo* experiments

**Fig 1:**
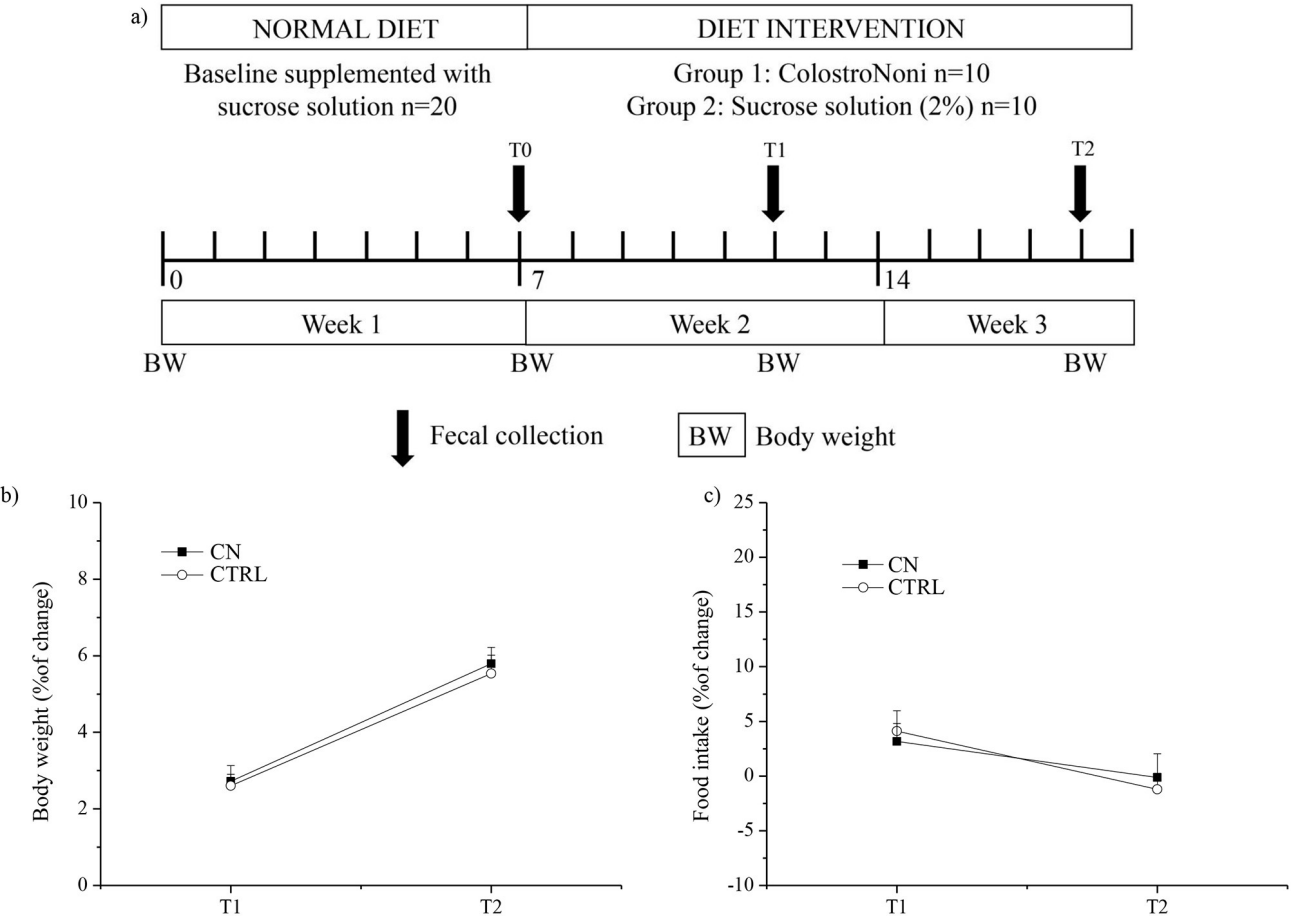
Timeline and results of the experimental procedure. Panel A shows the schedule of the experimental procedures. Panel B and C display the body weight and food intake percentage changes relative to the respective ‘T0’ values, during the experiment, respectively. Values are expressed as means ± standard error of mean (SEM).

The timeline of all procedures is illustrated in Fig 1. In detail, rats were housed individually in polymethyl methacrylate (Plexiglas) cages (39 cm × 23 cm × 15 cm). During the first week, animals continued to follow a normal chow diet but received daily oral administration of a 2% sucrose solution (volume: 500μl), in order to train them to drink spontaneously from a syringe. Subsequently, rats were randomized in two groups (n = 10 each) and were either fed with a standard diet supplemented with COLOSTRONONI (Product Number 934744602, Lot # A04268, Guna S.p.a., Milan, Italy) dissolved in sucrose solution (2%) (CN group) [0.500 gr/kg (w/w)], or maintained with a standard diet supplemented with sucrose solution (2%) (CTRL group) for the following 12 days. Finally, at the end of experimental protocol, the rats were euthanized. Furthermore, in order to evaluate the influence of COLOSTRONONI treatment in gut microbiota composition of rats we have collected the faecal samples at three different time points before (T0), during (T1) and after (T2) treatment (Fig 1). At the same time-points, BW was measured and BW changes were calculated as previously described [15].

### Faecal and cecal bacterial DNA extraction

Faecal samples were subjected to DNA extraction using the QIAmp DNA Stool Mini Kit following the manufacturer’s instructions (Qiagen). Furthermore, bacterial DNA extraction from cecal samples was performed using PowerViral Environmental RNA/DNA Isolation Kit Sample (Qiagen) following the protocol’s instruction. DNA concentration and purity were then determined with a Picodrop microlitre Spectrophotometer (Picodrop).

### 16S rRNA gene amplification and sequencing

Partial 16S rRNA gene sequences were amplified from extracted DNA using primer pair Probio_Uni and/Probio_Rev, which targets the V3 region of the 16S rRNA gene sequence [16]. Illumina adapter overhang nucleotide sequences are added to the partial 16S rRNA gene-specific amplicons, which were the further processed employing the 16S Metagenomic Sequencing Library Preparation Protocol (Part #15044223 Rev. B–Illumina; see also below). In accordance with the Illumina protocols, PCR products obtained following amplification of the 16S rRNA gene sequences were purified by magnetic purification step involving the Agencourt AMPure XP DNA purification beads (Beckman Coulter Genomics GmbH, Bernried, Germany) in order to remove primer dimers. DNA concentration of the amplified sequence library was estimated through fluorimetric Qubit quantification system (Life Technologies). Amplicons were diluted to 4 nM and 5 μl of each diluted DNA amplicons were mixed to prepare the pooled final library. Sequencing was performed using an Illumina MiSeq sequencer with MiSeq Reagent Kit v3 chemicals.

### 16S rRNA-microbial profiling analysis

Following sequencing, the. fastq files were processed using a custom script based on the QIIME software suite [17]. Paired-end reads pairs were assembled to reconstruct the complete Probio_Uni / Probio_Rev amplicons. Quality control retained sequences with a length between 140 and 400 bp and mean sequence quality score >20 while sequences with homopolymers >7 bp and mismatched primers were omitted. In order to calculate downstream diversity measures (alpha and beta diversity indices, Unifrac analysis), 16S rRNA Operational Taxonomic Units (OTUs) were defined at ≥ 99% sequence homology using uclust [18] and OTUs with less than 10 sequences were filtered. All reads were classified to the lowest possible taxonomic rank using QIIME [17] and a reference dataset from the SILVA database [19] for 16S rRNA data. Biodiversity of the samples (alpha-diversity) was calculated with Chao1 and Shannon indexes. Similarities between samples (beta-diversity) were calculated by weighted and unweighted uniFrac [20]. The range of similarities is calculated between the values 0 and 1. PCoA representations of beta-diversity were performed using QIIME [17].

### Statistical analyses

PERMANOVA analysis was performed, using QIIME [17] to evaluate the differences in microbiota composition between CN and CTRL group. All data were presented as means ± S.E.M. Comparison between the two groups were made using un-paired Student’s t-test. Observed differences corresponding to *P*<0.05, *P*<0.01 and *P<*0.001 were considered, respectively, statistically significant, highly significant or extremely highly significant. All analyses were performed using Prism 5 software (GraphPad Software Inc. San Diego, CA).

### RNA isolation and gene expression

Total RNA was isolated starting from 1 gr of caeca tissue that was suspended in 1 ml of QIAzoL (Qiagen, UK) and placed in a tube containing 0.8 g of glass beads (diameter, 106 μm; Sigma). The cells were lysed by shaking the mix on a BioSpec homogenizer at 4°C for 2 min (maximum setting). The mixture was then centrifuged at 12,000 rpm for 15 min, and the upper phase containing the RNA-containing sample was recovered. The RNA sample was further purified by RNeasy Mini Kit (QIAGEN) following the specific protocols. The quality of the RNA was checked by analysing the integrity of rRNA molecules by the MultiTape Station (Agilent). Reverse transcription to cDNA was performed with the iScript Select cDNA synthesis kit (Bio-Rad Laboratories) according to the supplier's instructions. The mRNA expression levels were analyzed with SYBR green technology in quantitative real-time PCR (qRT-PCR) using QPCR Green Master Mix LRox (Biotech Rabbit GmbH) on a Bio-Rad CFX96 system according to the manufacturer’s instructions (Bio-Rad). The primers used are indicated in S6 Table in the supplemental material. Quantitative PCR was carried out according to the following cycle: initial hold at 96°C for 30 s and then 42 cycles at 96°C for 15 s and Tm°C for 30 s. Statistical significance between means was analyzed using the unpaired Student t test with a threshold P < 0.05. Values are expressed as the means ± the SEMs of rats of each group. Statistical calculations were performed using the software program GraphPad Prism 5.

### Data deposition

Raw sequences of 16S rRNA gene profiling are accessible through SRA study accession numbers PRJNA497777.

## Results and discussion

### Animal growth and food intake

In order to evaluate if COLOSTRONONI is able to modulate rat physiological conditions, we performed an *in vivo* experiment using two groups of rats, one receiving COLOSTRONONI (CN) in their diet and the other maintained on a standard diet (CTRL). The effect of COLOSTRONONI on the body weight (BW), as well as on Food Intake (FI) of the animals was recorded (Fig 1).

Two-way ANOVA for repeated measures yielded a significant effect of time for BW (*F* = 232.09, *p*-value < 0.001) and FI/BW (*F* = 22.40, *p*-value < 0.001) changes. BW increased similarly in both groups during the experimental protocol. Likewise, no significant differences between the two groups were found in FI changes. Noteworthy, FI appears to increase only during the first week of CN/CTRL condition (CN: + 3.17%; CTRL: + 4.12%).

These data show that COLOSTRONONI treatment does not influence BW growth as well as FI in rats.

### Characterization of the effects of COLOSTRONONI on the gut microbiota composition of rats

**Fig 2:**
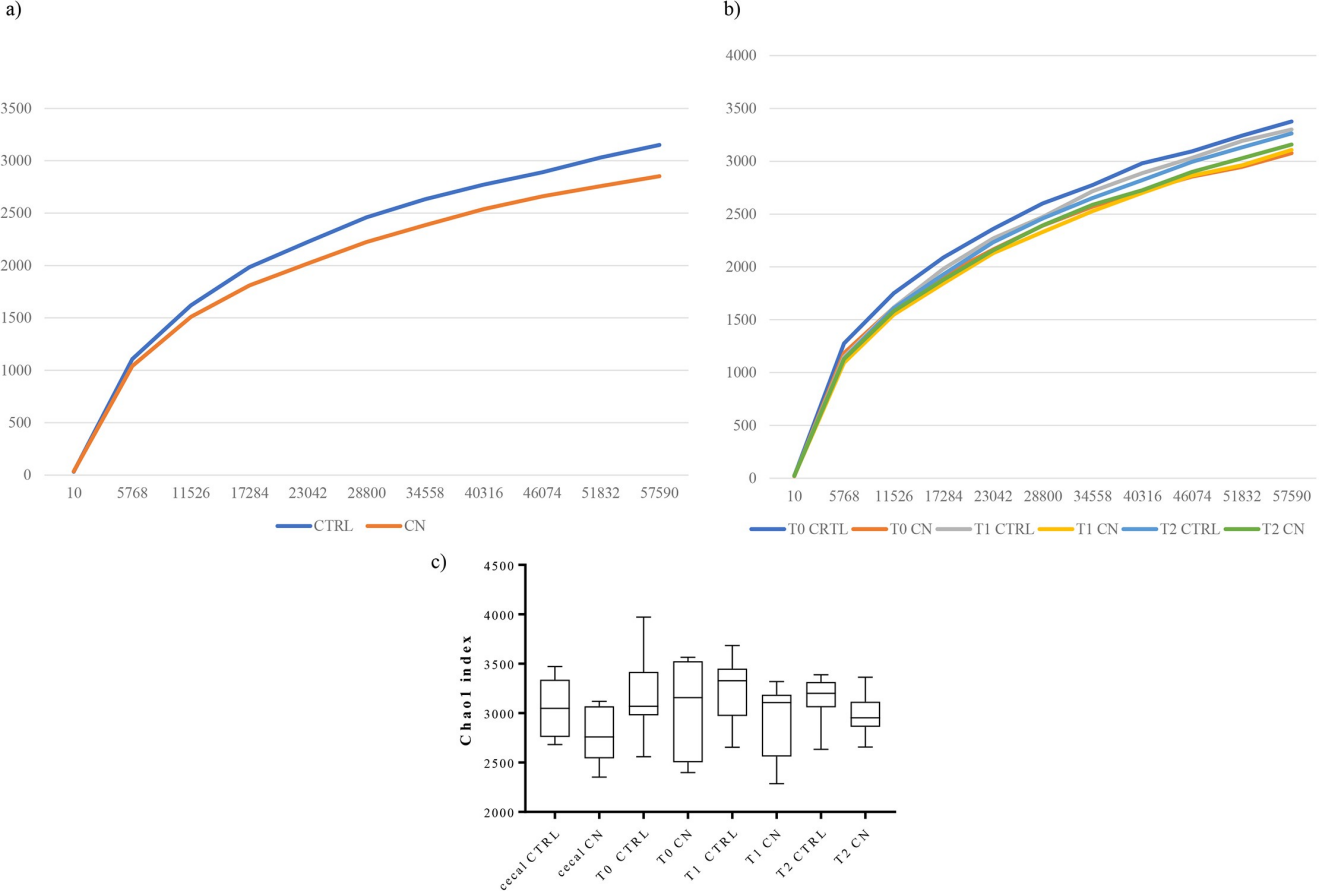
Alpha-diversity of CN and CTRL group. Panels a and b report average alpha-diversity obtained using Chao1 index for cecal and faecal samples, respectively. Panel c shows box and whisker plot. The bands inside the box are the median, the ends of the whisker represent the minimum and the maximum of all the data.

**Fig 3:**
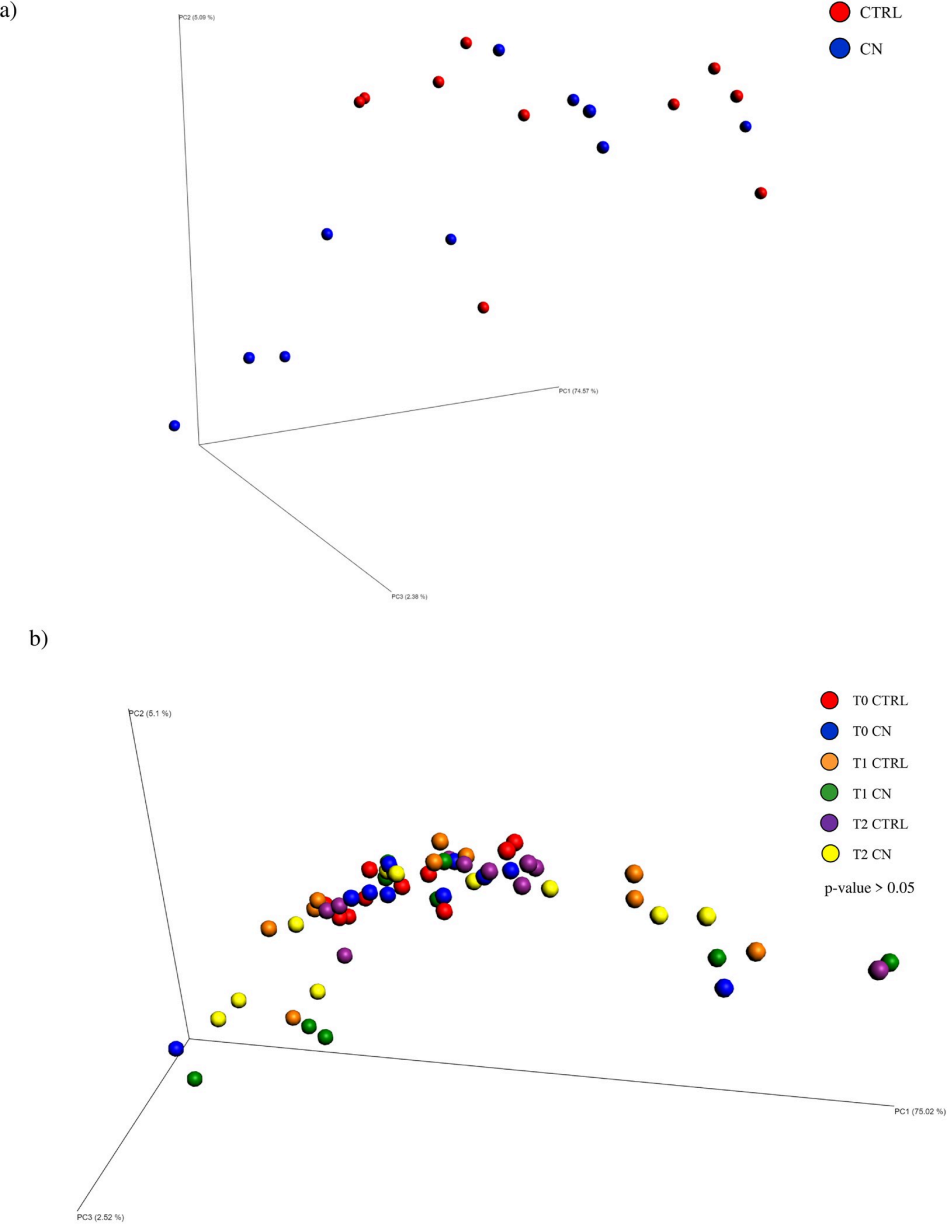
Beta diversity analysis of fecal and cecal samples. Panel a and b consists in a PCoA illustrating the beta-diversity of CN and CTRL cecal samples calculated through unweighted and weighted UniFrac, respectively. Panel c and d shows in a PCoA reporting the beta-diversity of CN and CTRL fecal samples calculated through unweighted and weighted UniFrac, respectively. It has also been reported the p-value calculated through a PERMANOVA analyses.

Fecal and caecal samples were used to determine the microbiota taxonomic profile associated with COLOSTRONONI dietary supplement or not by means of 16S rRNA-sequencing analysis as described previously [16]. Next Generation sequencing of the 80 samples, including fecal samples at different time points and caecal samples, produced a total of 5,727,788 reads, ranging from 43,050 to 92,936 per sample (S7 Table). Quality and chimera filtering led to a total of 4,863,323 filtered reads with an average of 60,791 and ranging from 27,534 to 82,880 reads per sample (S7 Table). Chao1 biodiversity curves suggesting that in all cases the obtained sequencing depth was appropriate to cover the large majority of the microbiota biodiversity contained in the samples (Fig 2A and 2B). Notably, CN supplementation was not associated with evident alterations and/or reduction of the biodiversity of the gut microbiota in the caecal samples as well as in fecal samples during all treatment periods (p -value > 0.05 calculated through un-paired Student’s t-test) (Fig 2C). In order to evaluate the rat microbiota differences between CN and CTRL groups, we analyzed the beta-diversity based on unweighted and weighted UniFrac for these groups, followed by Principal Coordinate Analysis (PCoA) (Fig 3). The PCoA analyses were performed on fecal samples at different time point as well as on the caecal samples, separately. These data highlight the occurrence, for both groups, of only one cluster (Fig 3), which suggest a maintenance of the stability of gut microbiota composition. Notably, these results were also validated by the PERMANOVA statistical analysis (p-value > 0.05) (Fig 3).

**Fig 4:**
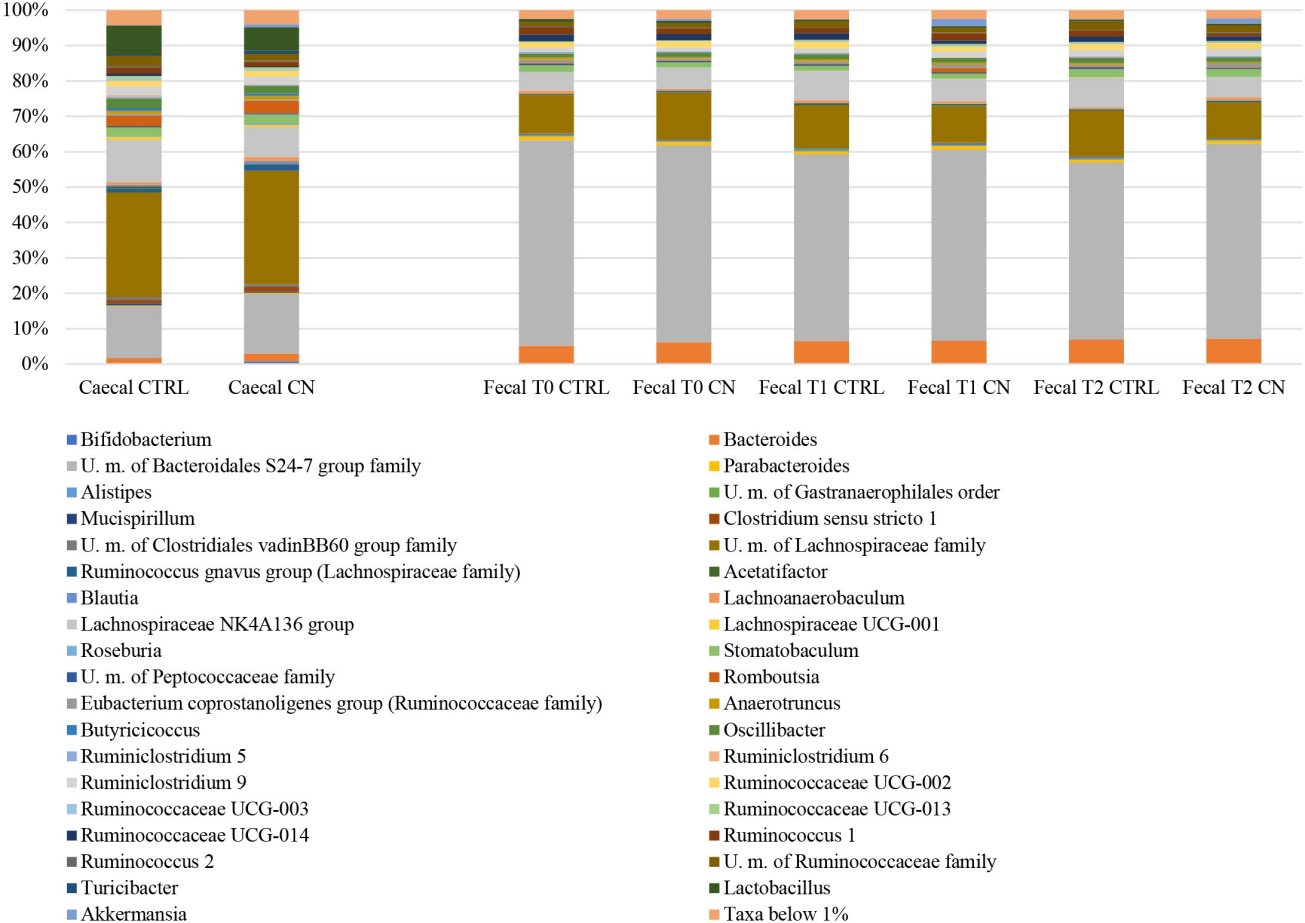
16S rRNA profiling of CN and CTRL rat cecal and fecal samples. Bar plots show the average of relative abundance variation in CN respect to CTRL rats at genus level. Only taxa with average relative abundance > 1% in these groups are shown.

**Fig 5:**
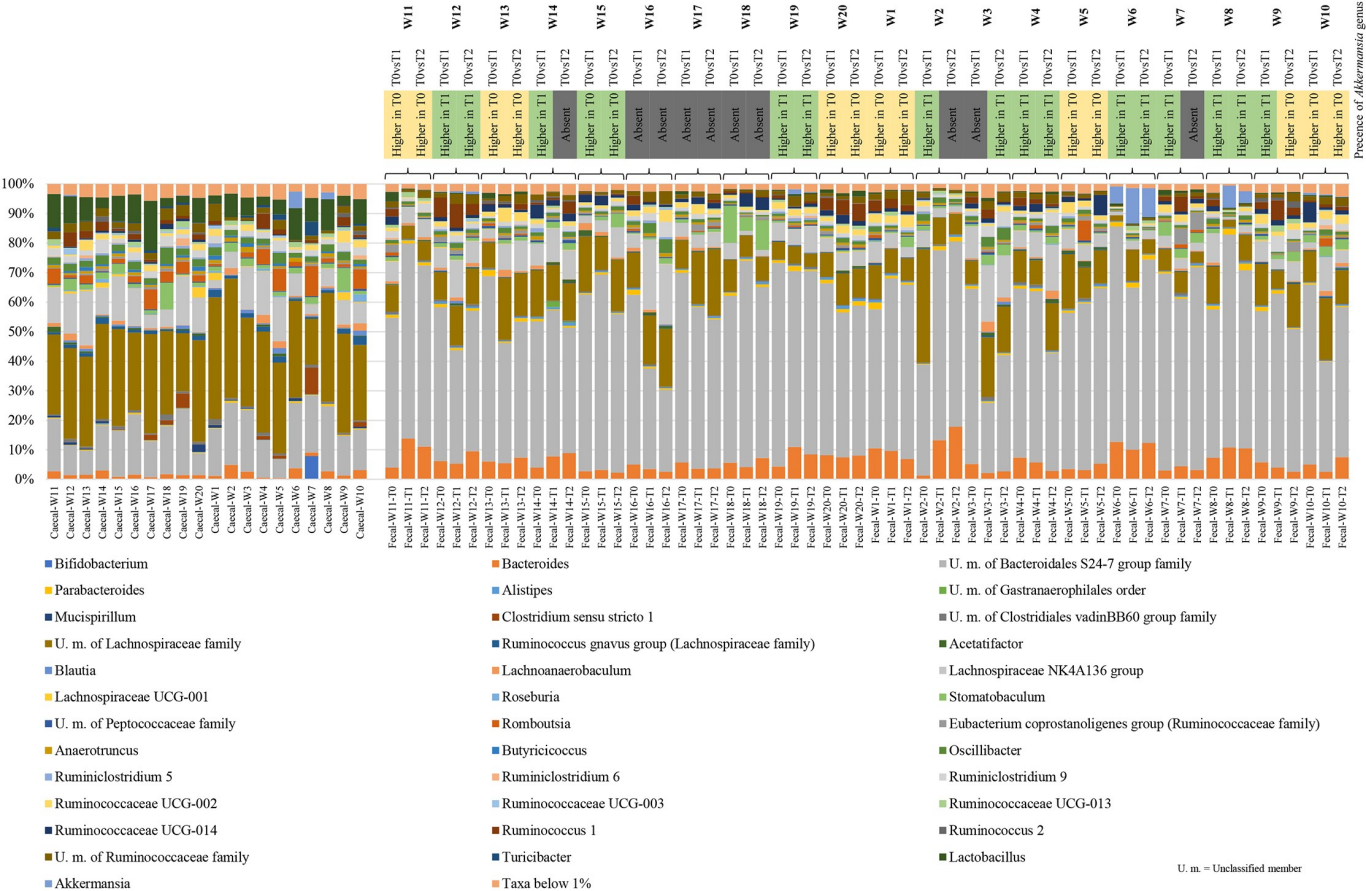
16S rRNA profiling of CN and CTRL rats cecal and fecal samples. Bar plot show the relative abundance variation in CN respect to CTRL rats at genus level for each animal. Only taxa with average relative abundance > 1% in these groups are shown. Heat map on the top of bar plot shows the increase/decrease of the genus *Akkermansia*.

The caecal microbiota composition of rats was shown to be dominated by the Firmicutes phylum (average value 79.92%), followed by Bacteroidetes (average values 18.07%). Conversely, the analysis of fecal microbiota composition highlighted a high abundance of Bacteroidetes (average value 62.27) and Firmicutes phylum (average values 36.61%). The most abundant bacterial taxa were Bacteroidales S24-7 group family (15.67% and 54.29% in caecal samples and in fecal samples, respectively) and two different genera belonging to Lachnospiraceae family (total average of 41.00% 18.14% in caecal samples and in fecal samples, respectively) (Figs 4 and 5). The comparison of the microbiota of caecal and fecal samples belonging to CN and CTRL rats, respectively, indicated no significant differences in the microbiota composition between each groups. These results support the hypothesis that COLOSTRONONI product does not affect (neither negatively nor positively) the gut microbiota homeostasis in healthy conditions, maintaining the equilibrium between the different bacterial taxa constituting the gut microbiota. Focusing on the genus *Akkermansia*, i.e., *Akkermansia muciniphila*, which was reported to exert health-promoting activities through the modulation of the host immune system and by the proliferation of anti-inflammatory regulatory T cells, we observed a slight increase of this genus in the CN treated fecal samples (relative abundance average of 0.66% ± 1.80%, 1.97% ± 4.20% and 1.39% ± 3.19% in T0, T1 and T2, respectively) compared with the control group (relative abundance average of 0.02% ± 0.05%, 0.22% ± 0.53% and 0.12% ± 0.30% in T0, T1 and T2, respectively) [21]. In detail, the prevalence obtained from the comparison of each sample T0 with the respective T1 or T2 sample in the CN group revealed an increase of this taxon in 50% of cases (6 samples T1 and 4 samples T2) (Fig 5). In contrast, the prevalence of the comparison of each sample T0 with the respective T1 or T2 sample in the CTRL group revealed an increase of this taxon in only 25% of cases (3 samples T1 and 2 samples T2) (Fig 5).

### Identification of molecular effects of COLOSTRONONI on the systemic inflammatory responses

In order to explore the impact of COLOSTRONONI on cytokines expression in rats, we evaluated the induction of genes encoding for IL-10, IL-12, IL-8 and TNF-α by using a qRT-PCR approach. In this context, we observed different cytokine expression between the rats supplemented with CN and the CTRL group [rats supplemented with sucrose solution (2%)].

The results of gene expression assays on healthy rat cecal tissue show that a significant reduction of IL-10, IL-12 and TNF-α expression levels was detectable in the CN group.Particularly interesting is the evaluation of the ratios between IL-10 and IL-12 and between TNF-α and IL-10.

**Fig 6:**
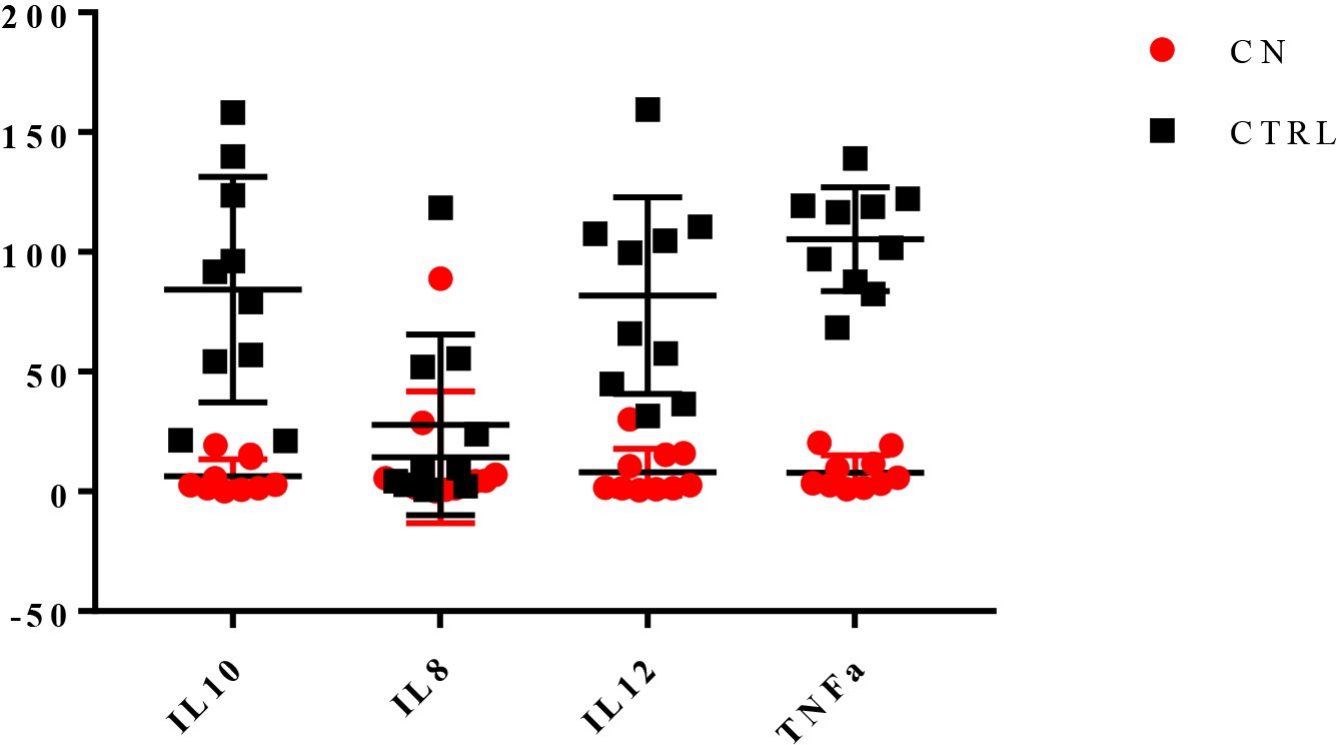
Evaluation of the expression of rat cytokines. Horizontal level represents the medians. The y axis represents the level of expression as normalized expression (ΔΔCt) according to CFX96 Bio-Rad software.

IL-10/IL-12 ratio (about 1:1, under normal conditions) [22, 23], is currently investigated as a marker for inflammatory diseases and/or dysbiosis. It is also useful for the evaluation of the effects of supplementation with probiotics (increased ratio due to increased expression of IL-10) [23, 24]. The highlighted simultaneous reduction of IL-12 and IL-10 (74 and 78 folds respectively) (Fig 6) preserves the normal IL-10/IL-12 ratio, in agreement with the lack of changes in the microbiota composition in the two experimental groups.

TNF-α/IL-10 ratio is reduced in CN group compared with the CTRL one; this result, due to the positive “delta” between TNF-α (-97 folds) and IL-10 gene expression reduction (23 folds in favor of IL-10) (Fig 6) may be considered as a marker of an anti-inflammatory activity of COLOSTRONONI.

Interestingly, COLSTRONONI seems to modulate inflammatory response by a refined regulation of both TNF-α and IL-10 gene expression instead of inducing the up-regulation of IL-10. The simultaneous reduction of TNF-α, IL-10, and IL-12 gene expression allow us to hypothesize that COLOSTRONONI in healthy subject plays an important role in immunomodulation mechanisms through a slight reduction of physiological inflammation (low TNF-α levels and reduced TNF-α/IL-10 ratio) preserving, at the same time, the physiological mechanism of immunotolerance (maintenance of IL-10/IL-12 ratio).

### Impact of COLOSTRONONI on cell permeability

The primary constituents in the transmembrane group include the claudins, occludin, and junctional adhesion molecules (JAMs) [25]. These several gut permeability markers were evaluated by qRT-PCR. These data highlighted no significant differences in the gene expression for claudins (claudin-1, claudin-2 and claudin-5), as well as JAM-A and occludin, in rats treated with COLOSTRONONI (CN group) respect to in non-treated rats (CTRL group). These data suggest that the supplementation of COLOSTRONONI could not influence the gut permeability of healthy rats. However, it is worth mentioning that under healthy conditions the level of expression of TJs genes is normally not modified.

## Conclusion

The evaluation of the potential dietary supplement features of COLOSTRONONI under *in vivo* conditions revealed the ability of this dietary supplement to preserve the gut microbiota climax as well as its influences on the mammalian host innate immunity. Furthermore, the COLOSTRONONI treatment provoked a slight increase of the genus *Akkermansia*. Interestingly, *A*. *muciniphila* is an intestinal bacterium, which has been proposed as a novel health-promoting bacterial species due to its immunomodulatory properties.

COLOSTRONONI also showed a significant lowering effect in the expression of genes coding for IL-10, Il-12 and TNF-α response which allows us to hypothesize an immunomodulatory activity of this dietary supplement. It is remarkable that TNF-α mediates changes in goblet cell expression and mucin sulfation, and that its production is inversely correlated with epithelial permeability [26, 27]. Interestingly, the reduction of TNF-α gene expression in the CN group probably improved the mucus synthesis., which consequently could promote a slight increase of *A*. *muciniphila*.

COLOSTRONONI does not influence the IL-8 expression; this result is in agreement with the observed maintenance of normal epithelial integrity, which is confirmed by the absence of modulation of genes enconding for claudins, occludin and JAMs, whose expression has been shown to be regulated in dose-dependent manner by IL-8 itself [27]. These results reinforce the concept that bovine colostrum and *M*. *citrifolia* influence the mammalian host innate immunity.

Future studies should be performed using different amounts of COLOSTRONONI in order to assess if the homeostatic role of this dietary supplements toward the mammalian gut microbiota is dose-dependent.

In addition, it will be interesting to evaluate if the homeostatic role of COLOSTRONONI on the gut microbiota is also exploited under acute inflammatory conditions as those occurring during Inflammatory Bowel Disease (IBD). In this context, a recent study involving an IBD murine model treated with bovine colostrum (BC) demonstrated an interesting protective role on the gut microbiota of animal driven by BC [28]. So far, we do not know if the here identified effects of COLOSTRONONI on the gut microbiota in healthy rats will be also valid in animals displaying IBD symptoms.

In this context, future *in vivo* clinical trials will be pivotal to confirm the role of COLOSTRONONI on gut microbiota composition in subjects with inflammatory bowel disease (IBD), such as ulcerative colitis and Crohn disease.

## Supporting information

S1 Fig Manufacturing process flow sheet: COLOSTRONONI 2205C02. (JPG)

S1 Text NC3Rs ARRIVE guidelines checklist. (PDF)

S1 Table List of ingredients of COLOSTRONONI. (DOCX)

S2 Table Product quality control and standardization of COLOSTRONONI. (DOCX)

S3 Table Stability studies of COLOSTRONONI. (DOCX)

S4 Table Chemical characterization of bovine colostrum. (DOCX)

S5 Table *Morinda citrifolia L*. powdered juice specifications. (DOCX)

S6 Table Primers used in this study. (DOCX)

S7 Table Filtering table of the analyzed samples. (DOCX)
