# Supplementary figures and images for: Exploring the effects of COLOSTRONONI on the mammalian gut microbiota composition
Source: PLoS One. 2019 May 31;14(5):e0217609. doi: 10.1371/journal.pone.0217609 (PMC6544264; doi:10.1371/journal.pone.0217609)

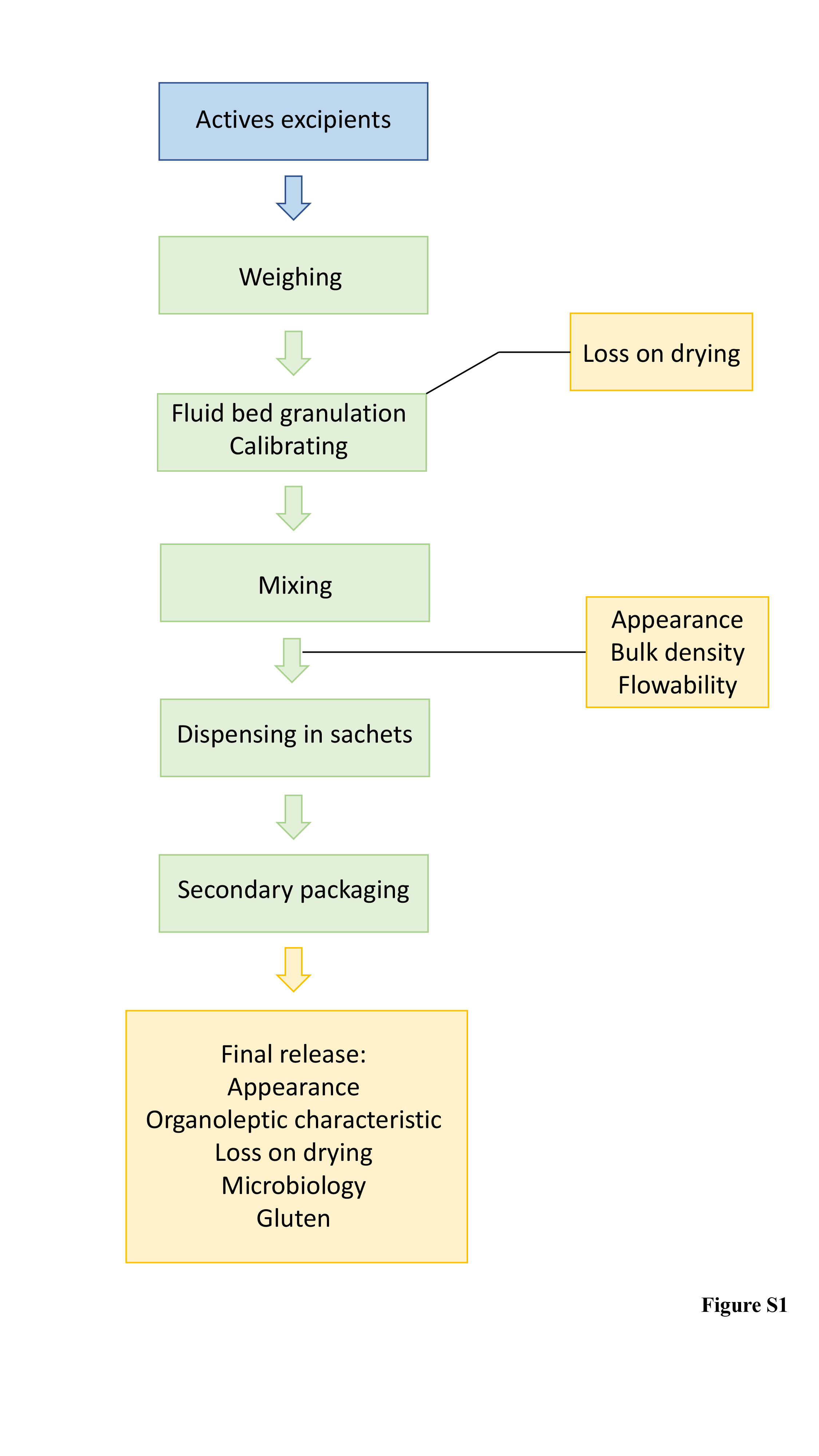

Supplement: S1 Fig — (JPG) [file pone.0217609.s001.jpg]
