# Supplementary material for: Exploring the effects of COLOSTRONONI on the mammalian gut microbiota composition
Source: PLoS One. 2019 May 31;14(5):e0217609. doi: 10.1371/journal.pone.0217609 (PMC6544264; doi:10.1371/journal.pone.0217609)
Supplement: S1 Table — (DOCX) [file pone.0217609.s003.docx]

**Table S1**. List of ingredients of COLOSTRONONI.

| **Ingredients** | **Mg/bst** | **%** |
| --- | --- | --- |
| Sweetener : sorbitol | 930,800 | 51,711 |
| Lyophilized bovine colostrum | 550,000 | 30,556 |
| Noni (*Morinda citrifolia* L) fruit juice powered | 200,000 | 11,111 |
| Maltodextrin | 75,000 | 4,167 |
| Flavor | 40,000 | 2,222 |
| Anticaking agent: silicon dioxide | 3,000 | 0,167 |
| Sweetener : sucralose | 1,200 | 0.067 |

| **Active Ingredients** | **Quantity per Dose (1,8 g sachet)** |
| --- | --- |
| Bovine colostrum | 550 mg |
| *Morinda citrofolia* fruit juice powdered | 200 mg |
