# Supplementary material for: Exploring the effects of COLOSTRONONI on the mammalian gut microbiota composition
Source: PLoS One. 2019 May 31;14(5):e0217609. doi: 10.1371/journal.pone.0217609 (PMC6544264; doi:10.1371/journal.pone.0217609)
Supplement: S2 Table — (DOCX) [file pone.0217609.s004.docx]

**Table S2.** Product quality control and standardization of COLOSTRONONI.

| **Test** | **Unit** | **Limits** | **Result** |
| --- | --- | --- | --- |
| Appearance | Free flowing light-yellow powder | | Complies |
| Loss on drying (75°C/c.w.) | % | 2,0 | 0.8 |
| Organoleptic features | Cream-biscuit taste | | Complies |
| Gluten | Ppm | 20,0 | <5,2 |
| TAMC | ufc/g | 10000 | <1000 |
| TYMC | ufc/g | 100 | <10 |
| Enterobacteriacee | ufc/g | 100 | <10 |
| TAMC: Total Anaerobic Microbial Count; TYMC: Total Yeast and Mold Count | | | |
