# Supplementary material for: Exploring the effects of COLOSTRONONI on the mammalian gut microbiota composition
Source: PLoS One. 2019 May 31;14(5):e0217609. doi: 10.1371/journal.pone.0217609 (PMC6544264; doi:10.1371/journal.pone.0217609)
Supplement: S3 Table — (DOCX) [file pone.0217609.s005.docx]

**Table S3.** Stability studies of COLOSTRONONI.

| **Determinations** | **Specifications** | **Initial**  **18-06-2008** | **1 Month**  **18-06-2008** | **2 Month**  **18-06-2008** | **3 Month**  **18-06-2008** |
| --- | --- | --- | --- | --- | --- |
| Appearance | Free flowing light-yellow powder | Complies | Complies | Complies | Complies |
| Loss on drying (75°C/c.w.) | < 2 % | 1,30 % | 1,45 % | 1,51 % | 1,60 % |
| Organoleptic features | Cream-biscuit taste | Complies | Complies | Complies | Complies |
| Gluten | 20,0 Ppm | Complies | Complies | Complies | Complies |
| TAMC | 10000 ufc/g | Complies | Complies | Complies | Complies |
| TYMC | 100 ufc/g | Complies | Complies | Complies | Complies |
| Enterobacteriacee | 100 ufc/g | Complies | Complies | Complies | Complies |
| TAMC: Total Anaerobic Microbial Count; TYMC: Total Yeast and Mold Count | | | | | |
