# Supplementary material for: Exploring the effects of COLOSTRONONI on the mammalian gut microbiota composition
Source: PLoS One. 2019 May 31;14(5):e0217609. doi: 10.1371/journal.pone.0217609 (PMC6544264; doi:10.1371/journal.pone.0217609)
Supplement: S4 Table — (DOCX) [file pone.0217609.s006.docx]

**Table S4.** Chemical characterization of bovine colostrum.

| **Tests** | **Specifications** | **Result** |
| --- | --- | --- |
| Appearance | Milled, fine, creamish powder | Complies |
| Color |  | Complies |
| Smell | Slightly milky to odorless | Complies |
| pH | 5,00 - 7,00 | 6,40 |
| Water | < 6% | 3,70 |
| Total ash | < 8% | 6,50 |
| Proteins | > 60% | 67,90 |
| Fats | < 4% | 2,60 |
| Carbohydrates | < 25% | 19,30 |
| Total Immunoglobulins | > 35% | 36,40 |
| Immunoglobulins type G | > 30% | 32,30 |
| Lactoferrin LFL | > 3% | > 3 |
| Proline-rich polypeptides-PRP | > 2% | > 2 |
| Lead | < 0,02 ppm | < 0,02 |
| Cadmium | < 0,1 ppm | < 0,1 |
| Mercury | < 0,1 ppm | < 0,1 |
| Arsenic | < 0,5 ppm | < 0,5 |
| Dioxins | < 2,5 pg/gr | Complies |
| Dioxins and dioxin-like PCB’s | < 4,5 pg/gr | Complies |
| Melamine | < 0,5 ppm | Complies |
| Aflatoxin M1 | < 0,05 ppb | Complies |
| Inhibitor substances | Negative | Complies |
| Total aerobic count | < 10000 CFU/gr | 8300 |
| Yeasts and molds | < 500 CFU/gr | 150 |
| Enterobacteriaceae | < 10 CFU/gr | < 10 |
| *Escherichia coli* | Negative | Complies |
| *Salmonella* | Negative | Complies |
| *Staphylococcus aureus* | Negative | Complies |
| *Listeria monocytogenes* | Negative | Complies |
