# Supplementary material for: Exploring the effects of COLOSTRONONI on the mammalian gut microbiota composition
Source: PLoS One. 2019 May 31;14(5):e0217609. doi: 10.1371/journal.pone.0217609 (PMC6544264; doi:10.1371/journal.pone.0217609)
Supplement: S5 Table — (DOCX) [file pone.0217609.s007.docx]

**Table S5.** *Morinda citrifolia L.* powdered juice specifications.

| **Compounds** | **Specifications** | **Result** |
| --- | --- | --- |
| Moisture | 89 - 93% | 48 - 53% |
| Protein | < 0,6 g/100 g | 3 - 3,5 g/100 g |
| Fat | < 0,2 g/100 g | < 0,04 g/100 g |
| Ash | < 1 g/100 g | 4,5 - 5 g/100 g |
| Total carbohydrates | 5 - 10 g/100 g | 37 - 45 g/100 g |
| Fructose | 0,5 - 2 g/100 g | 9 - 11 g/100 g |
| Glucose | 0,5 - 2 g/100 g | 9 - 11 g/100 g |
| Dietary Fiber | 1,5 - 3 g/100 g | 1,5 - 5 g/100 g |
| 5,15-dimethylmorindol | 0,19 – 0,20 µg/100 mL | 0,191– 0,77 µg/100 mL |
| Lucidin | Not detectable | Not detectable |
| Alizarin | Not detectable | Not detectable |
| Rubiadin | Not detectable | Not detectable |
