# Supplementary material for: Exploring the effects of COLOSTRONONI on the mammalian gut microbiota composition
Source: PLoS One. 2019 May 31;14(5):e0217609. doi: 10.1371/journal.pone.0217609 (PMC6544264; doi:10.1371/journal.pone.0217609)
Supplement: S6 Table — (DOCX) [file pone.0217609.s008.docx]

**Table S6**. Primers used in this study.

| **Name** | **Sequence** | |
| --- | --- | --- |
| **Occludin** | **Fw** | 5’-CCTCCAATGGCAAAGTGAAT-3’ |
|  | **Rev** | 5’-CTCCCCACCTGTCGTGTAGT-3’ |
| **Claudin-1** | **Fw** | 5'-CTGGGTTTCATCCTGGCTTC-3' |
|  | **Rev** | 5'-TTGATGGGGGTCAAGGGGTC-3' |
| **Claudin-2** | **Fw** | 5'-TTAGCCCTGACCGAGAAAGA-3' |
|  | **Rev** | 5'-AAAGGACCTCTCTGGTGCTG-3' |
| **Claudin-5** | **Fw** | 5'-TGGAACGCTCAGATTTCATC-3' |
|  | **Rev** | 5'-AGGAAGGCAACCCCTCTAAG-3' |
| **JAM-A** | **Fw** | 5'-CTGATCTTTGACCCCGTGAC-3' |
|  | **Rev** | 5'-ACCAGACGCCAAAAATCAAG-3' |
| **IL10** | **Fw** | 5'-GCCCAGAAATCAAGGAGCAT-3' |
|  | **Rev** | 5'-TGTAGACACCTTGGTCTTGGAG-3' |
| **IL8** | **Fw** | 5'-TCATAGCCACTCTCAAGGG-3' |
|  | **Rev** | 5'-GCCTTGCCTTTGTTCAGT-3' |
| **IL12** | **Fw** | 5'-TGTCTTAGCCAGTCCCGAAA-3' |
|  | **Rev** | 5'-TGATCGATGTCTTCAGCAGTG-3' |
| **TNF-α** | **Fw** | 5'-CTTCTGTCTACTGAACTTCGGG-3' |
|  | **Rev** | 5'-CAGGCTTGTCACTCGAATTTTG-3' |
| **18S** | **Fw** | 5’- GTGATCCCTGAGAAGTTCCAG -3’ |
|  | **Rev** | 5’-TCGATGTCTGCTTTCCTCAAC-3’ |
| **mGAPDH** | **Fw** | 5’- CTTCACCACCATGGAGAAGGC -3’ |
|  | **Rev** | 5’- GGCATGGACTGTGGTCATGAG -3’ |
| **mHspd1** | **Fw** | 5’- GCAGTGTTTGGAGAAGAGGG -3’ |
|  | **Rev** | 5’- GGTGACAATGACCTCCCCA -3’ |
