# Supplementary material for: Exploring the effects of COLOSTRONONI on the mammalian gut microbiota composition
Source: PLoS One. 2019 May 31;14(5):e0217609. doi: 10.1371/journal.pone.0217609 (PMC6544264; doi:10.1371/journal.pone.0217609)
Supplement: S7 Table — (DOCX) [file pone.0217609.s009.docx]

**Table S7.** Filtering table of the analyzed samples.

| **Sample** | | **Number of sequenced pe reads** | **Number of pe reads with mean quality > 20** | **Number of merged pe reads** | **Human sequences** | **Ambiguous bases** | **Homopolymers > 7** | **Mismatch in primers >1** | **Reverse primer not found** | **Final Read Number** |
| --- | --- | --- | --- | --- | --- | --- | --- | --- | --- | --- |
| **Caecal CN** | **W01** | 57642 | 54194 | 51677 | 2926 | 1 | 6 | 1264 | 100 | 47380 |
| **Caecal CN** | **W02** | 75239 | 71996 | 68333 | 767 | 2 | 3 | 1848 | 126 | 65587 |
| **Caecal CN** | **W03** | 91469 | 79427 | 75511 | 6046 | 0 | 9 | 1754 | 129 | 67572 |
| **Caecal CN** | **W04** | 92610 | 83700 | 79159 | 4331 | 0 | 8 | 2343 | 183 | 72294 |
| **Caecal CN** | **W05** | 73994 | 68220 | 65321 | 2013 | 0 | 3 | 1577 | 163 | 61565 |
| **Caecal CN** | **W06** | 73995 | 63102 | 59268 | 5495 | 0 | 9 | 1884 | 124 | 51756 |
| **Caecal CN** | **W07** | 67855 | 60908 | 57443 | 8121 | 0 | 3 | 1438 | 96 | 47785 |
| **Caecal CN** | **W08** | 72741 | 63835 | 60467 | 4167 | 0 | 6 | 1543 | 122 | 54629 |
| **Caecal CN** | **W09** | 70413 | 67497 | 64668 | 803 | 0 | 4 | 1723 | 86 | 62052 |
| **Caecal CN** | **W10** | 83037 | 77792 | 74845 | 2915 | 0 | 1 | 2071 | 82 | 69776 |
| **Caecal CTRL** | **W11** | 76761 | 74155 | 71564 | 6542 | 0 | 5 | 1517 | 94 | 63406 |
| **Caecal CTRL** | **W12** | 64606 | 62256 | 59753 | 2124 | 0 | 2 | 1801 | 86 | 55740 |
| **Caecal CTRL** | **W13** | 78485 | 75435 | 73196 | 8331 | 0 | 10 | 1577 | 93 | 63185 |
| **Caecal CTRL** | **W14** | 75979 | 73024 | 70047 | 2194 | 0 | 4 | 2300 | 88 | 65461 |
| **Caecal CTRL** | **W15** | 73874 | 71379 | 68337 | 1826 | 0 | 6 | 1916 | 80 | 64509 |
| **Caecal CTRL** | **W16** | 85471 | 82702 | 79658 | 3286 | 0 | 4 | 2083 | 95 | 74190 |
| **Caecal CTRL** | **W17** | 76577 | 72371 | 68834 | 4421 | 2 | 7 | 1743 | 117 | 62544 |
| **Caecal CTRL** | **W18** | 84762 | 80030 | 76195 | 6797 | 0 | 5 | 1761 | 103 | 67529 |
| **Caecal CTRL** | **W19** | 92271 | 86304 | 82046 | 3103 | 0 | 10 | 1912 | 152 | 76869 |
| **Caecal CTRL** | **W20** | 92936 | 83024 | 78407 | 6983 | 0 | 6 | 2145 | 158 | 69115 |
| **Fecal T0 CN** | **W01** | 65762 | 56271 | 52665 | 18 | 1 | 28 | 1898 | 82 | 50638 |
| **Fecal T0 CN** | **W02** | 71885 | 66213 | 61545 | 12 | 0 | 29 | 1976 | 135 | 59393 |
| **Fecal T0 CN** | **W03** | 77241 | 70998 | 66987 | 11 | 1 | 45 | 1938 | 128 | 64864 |
| **Fecal T0 CN** | **W04** | 63415 | 49561 | 46893 | 1 | 0 | 21 | 1266 | 78 | 45527 |
| **Fecal T0 CN** | **W05** | 80889 | 77396 | 73281 | 4 | 3 | 8 | 1768 | 131 | 71367 |
| **Fecal T0 CN** | **W06** | 64811 | 59035 | 55962 | 2 | 1 | 39 | 1503 | 133 | 54284 |
| **Fecal T0 CN** | **W07** | 48075 | 37387 | 35071 | 6 | 0 | 19 | 1101 | 101 | 33844 |
| **Fecal T0 CN** | **W08** | 43050 | 29857 | 28395 | 6 | 0 | 16 | 779 | 60 | 27534 |
| **Fecal T0 CN** | **W09** | 88225 | 84607 | 79584 | 11 | 1 | 11 | 2423 | 148 | 76990 |
| **Fecal T0 CN** | **W10** | 44616 | 42584 | 39964 | 2 | 1 | 7 | 1038 | 77 | 38839 |
| **Fecal T0 CTRL** | **W11** | 53802 | 47208 | 44570 | 5 | 0 | 24 | 1240 | 113 | 43188 |
| **Fecal T0 CTRL** | **W12** | 59862 | 50499 | 47102 | 4 | 0 | 23 | 1214 | 166 | 45695 |
| **Fecal T0 CTRL** | **W13** | 91591 | 84266 | 78472 | 24 | 0 | 27 | 1832 | 264 | 76325 |
| **Fecal T0 CTRL** | **W14** | 52108 | 37827 | 35174 | 2 | 0 | 23 | 860 | 146 | 34143 |
| **Fecal T0 CTRL** | **W15** | 74815 | 66880 | 61977 | 10 | 1 | 45 | 1799 | 279 | 59843 |
| **Fecal T0 CTRL** | **W16** | 62550 | 51832 | 48689 | 29 | 1 | 22 | 1242 | 163 | 47232 |
| **Fecal T0 CTRL** | **W17** | 74836 | 56335 | 52027 | 5 | 1 | 39 | 1666 | 180 | 50136 |
| **Fecal T0 CTRL** | **W18** | 66603 | 59494 | 55040 | 3 | 1 | 32 | 1692 | 153 | 53159 |
| **Fecal T0 CTRL** | **W19** | 55332 | 46579 | 43293 | 6 | 0 | 28 | 1175 | 161 | 41923 |
| **Fecal T0 CTRL** | **W20** | 48857 | 34566 | 33188 | 5 | 0 | 24 | 900 | 28 | 32231 |
| **Fecal T1 CN** | **W01** | 77523 | 74777 | 71331 | 10 | 0 | 8 | 1865 | 71 | 69377 |
| **Fecal T1 CN** | **W02** | 63047 | 61115 | 57903 | 5 | 1 | 10 | 1468 | 68 | 56351 |
| **Fecal T1 CN** | **W03** | 92007 | 88717 | 85022 | 5 | 2 | 7 | 2009 | 119 | 82880 |
| **Fecal T1 CN** | **W04** | 85564 | 81174 | 77178 | 33 | 0 | 12 | 2325 | 105 | 74703 |
| **Fecal T1 CN** | **W05** | 82120 | 77448 | 74402 | 4 | 1 | 14 | 1815 | 114 | 72454 |
| **Fecal T1 CN** | **W06** | 62173 | 59235 | 56130 | 5 | 0 | 6 | 1815 | 52 | 54252 |
| **Fecal T1 CN** | **W07** | 91221 | 88935 | 84514 | 11 | 0 | 8 | 2477 | 88 | 81930 |
| **Fecal T1 CN** | **W08** | 75293 | 71916 | 68385 | 1 | 0 | 9 | 1882 | 84 | 66409 |
| **Fecal T1 CN** | **W09** | 62624 | 60128 | 57848 | 2 | 0 | 9 | 1484 | 65 | 56288 |
| **Fecal T1 CN** | **W10** | 75423 | 73375 | 69649 | 4 | 1 | 9 | 1891 | 161 | 67583 |
| **Fecal T1 CTRL** | **W11** | 75049 | 71559 | 68845 | 20 | 0 | 11 | 1666 | 79 | 67069 |
| **Fecal T1 CTRL** | **W12** | 67414 | 64844 | 62250 | 10 | 0 | 8 | 1838 | 81 | 60313 |
| **Fecal T1 CTRL** | **W13** | 66884 | 63264 | 61292 | 5 | 1 | 9 | 1521 | 62 | 59694 |
| **Fecal T1 CTRL** | **W14** | 74169 | 72280 | 69215 | 9 | 1 | 13 | 2308 | 80 | 66804 |
| **Fecal T1 CTRL** | **W15** | 57361 | 55649 | 53146 | 0 | 0 | 13 | 1540 | 44 | 51549 |
| **Fecal T1 CTRL** | **W16** | 69036 | 66633 | 64035 | 1 | 0 | 9 | 1679 | 84 | 62262 |
| **Fecal T1 CTRL** | **W17** | 76119 | 73939 | 70624 | 10 | 0 | 9 | 1826 | 116 | 68663 |
| **Fecal T1 CTRL** | **W18** | 74777 | 73519 | 69935 | 3 | 1 | 9 | 1908 | 82 | 67932 |
| **Fecal T1 CTRL** | **W19** | 86235 | 83318 | 79507 | 5 | 0 | 13 | 1896 | 133 | 77460 |
| **Fecal T1 CTRL** | **W20** | 43992 | 43152 | 40949 | 3 | 0 | 12 | 1589 | 34 | 39311 |
| **Fecal T2 CN** | **W01** | 58822 | 57783 | 55394 | 2 | 1 | 7 | 1361 | 102 | 53921 |
| **Fecal T2 CN** | **W02** | 67927 | 66120 | 62465 | 2 | 0 | 4 | 2299 | 96 | 60064 |
| **Fecal T2 CN** | **W03** | 71084 | 69743 | 66195 | 0 | 1 | 10 | 1905 | 115 | 64164 |
| **Fecal T2 CN** | **W04** | 67977 | 64742 | 61451 | 2 | 1 | 9 | 1807 | 94 | 59538 |
| **Fecal T2 CN** | **W05** | 86502 | 84166 | 80993 | 6 | 1 | 14 | 2133 | 98 | 78741 |
| **Fecal T2 CN** | **W06** | 70147 | 68521 | 65635 | 0 | 0 | 10 | 1777 | 71 | 63777 |
| **Fecal T2 CN** | **W07** | 74425 | 73433 | 70648 | 3 | 0 | 11 | 1780 | 93 | 68761 |
| **Fecal T2 CN** | **W08** | 74416 | 73205 | 70089 | 3 | 1 | 15 | 2163 | 99 | 67808 |
| **Fecal T2 CN** | **W09** | 77164 | 76045 | 73541 | 4 | 0 | 9 | 1839 | 89 | 71600 |
| **Fecal T2 CN** | **W10** | 75907 | 74417 | 71070 | 6 | 0 | 9 | 2359 | 83 | 68613 |
| **Fecal T2 CTRL** | **W11** | 72421 | 70947 | 67551 | 18 | 0 | 16 | 2039 | 70 | 65408 |
| **Fecal T2 CTRL** | **W12** | 72727 | 71620 | 68901 | 5 | 0 | 13 | 1898 | 80 | 66905 |
| **Fecal T2 CTRL** | **W13** | 72029 | 70577 | 67544 | 2 | 0 | 4 | 1795 | 90 | 65653 |
| **Fecal T2 CTRL** | **W14** | 74559 | 73341 | 70039 | 3 | 0 | 13 | 1942 | 73 | 68008 |
| **Fecal T2 CTRL** | **W15** | 67751 | 66477 | 63630 | 0 | 0 | 8 | 1652 | 69 | 61901 |
| **Fecal T2 CTRL** | **W16** | 61585 | 60581 | 57525 | 1 | 0 | 11 | 1972 | 55 | 55486 |
| **Fecal T2 CTRL** | **W17** | 67764 | 66594 | 63947 | 4 | 1 | 9 | 1480 | 81 | 62372 |
| **Fecal T2 CTRL** | **W18** | 64960 | 63744 | 60496 | 0 | 0 | 10 | 1913 | 63 | 58510 |
| **Fecal T2 CTRL** | **W19** | 68864 | 67041 | 63645 | 6 | 1 | 7 | 1901 | 68 | 61662 |
| **Fecal T2 CTRL** | **W20** | 73684 | 72240 | 69016 | 2 | 1 | 9 | 1876 | 80 | 67048 |
